# Areas within the United States at the Highest Risk for African Swine Fever, Classical Swine Fever, and Foot-and-Mouth Disease Introduction

**DOI:** 10.1155/2023/8892037

**Published:** 2023-05-25

**Authors:** Sophie C. McKee, Vienna R. Brown, Stephanie A. Shwiff, Glorianna M. Giallombardo, Ryan S. Miller

**Affiliations:** ^1^Department of Economics, Colorado State University, Fort Collins, CO, USA; ^2^National Wildlife Research Center, United States Department of Agriculture, Animal and Plant Health Inspection Service, Fort Collins, CO, USA; ^3^National Feral Swine Damage Management Program, United States Department of Agriculture, Animal and Plant Health Inspection Service, Fort Collins, CO, USA; ^4^Virginia-Maryland College of Veterinary Medicine, Blacksburg, VA, USA; ^5^Center for Epidemiology and Animal Health, United States Department of Agriculture, Animal and Plant Health Inspection Service, Veterinary Services, Fort Collins, CO, USA

## Abstract

Domestic livestock production is a major component of the agricultural sector, contributing to food security and human health and nutrition and serving as the economic livelihood for millions worldwide. The impact of disease on global systems and processes cannot be understated, as illustrated by the effects of the COVID-19 global pandemic through economic and social system shocks and food system disruptions. This study outlines a method to identify the most likely sites of introduction into the United States for three of the most concerning foreign animal diseases: African swine fever (ASF), classical swine fever (CSF), and foot-and-mouth disease (FMD). We first created an index measuring the amount of potentially contaminated meat products entering the regions of interest using the most recently available Agricultural Quarantine Inspection Monitoring (AQIM) air passenger inspection dataset, the AQIM USPS/foreign mail, and the targeted USPS/foreign mail interception datasets. The risk of introduction of a given virus was then estimated using this index, as well as the density of operations of the livestock species and the likelihood of infected material contaminating the local herds. Using the most recently available version of the datasets, the most likely places of introduction for ASF and CSF were identified to be in central Florida, while FMD was estimated to have been most likely introduced to swine in western California and to cattle in northeastern Texas. The method illustrated in this study is important as it may provide insights on risk and can be used to guide surveillance activities and optimize the use of limited resources to combat the establishment of these diseases in the U.S.

## 1. Introduction

Transboundary animal diseases [1] have global economic impacts [2] and significant financial implications for countries experiencing outbreaks. Globalization and climate change make it likely that agricultural diseases will emerge in new locations with greater frequency [3]. High-density production systems, expansive global markets for animal products, and increasing livestock-wildlife interactions due to urbanization all increase the likelihood that a foreign animal disease (FAD) will be introduced into a country [4, 5]. Animal disease surveillance systems are intended to detect introductions of FADs into animal populations with the goal of early identification to minimize outbreak severity and potential economic impacts [6]. A primary challenge in developing surveillance systems is targeting surveillance at those populations at greatest risk of introduction. While studies are frequently conducted evaluating potential risk factors [7–13], the geographic extent of risk is less studied [14].

There are 118 FADs that are considered reportable by the World Organisation for Animal Health (WOAH). Each of these FADs has the potential to cause significant harm to the U.S. economy through morbidity, mortality, decreases in consumer demand [15], and a moratorium on international trade [16]. Among these diseases, at least 79% have a potential wildlife host that contributes to spread, complicating control, and three of them, African swine fever (ASF), classical swine fever (CSF), and foot-and-mouth disease (FMD), are among the most concerning [8, 17, 18]. ASF is a highly contagious viral disease of pigs, whose mortality rate can reach 100%. It is not a danger to human health, but it has devastating effects on pig populations and the farming economy [19]. Globally, since 2021 and as of January 05, 2023, ASF has been reported in five different world regions in 40 countries [19]. Currently, only control and eradication measures based mainly on early detection and strict stamping-out policies are available, as there is no viable vaccine against ASF [20]. CSF is a highly contagious and economically significant viral disease in pigs. The severity of the illness varies with the strain of the virus, the age of the pig, and the immune status of the herd. CSF is found in Central and South America, Europe, Asia, and parts of Africa. North America, Australia, and New Zealand are currently free of the disease [21]. FMD is a severe, highly contagious viral disease of livestock that has a significant economic impact. The disease affects cattle, swine, sheep, goats, and other cloven-hoofed animals. Foot-and-mouth disease is endemic in several parts of Asia, most of Africa, and the Middle East. In Latin America, the majority of countries apply zoning and are recognized as FMD-free, either with or without vaccination. Australia, New Zealand, Indonesia, Central and North America, and continental Western Europe are currently free of FMD [22].

These diseases are of particular economic concern for the US because the introduction of one of these FADs into the US could have global economic impacts, in particular through trade restrictions [23]. The US has the largest fed cattle industry in the world and is the world's largest producer of beef for domestic and export use [24]. The US is also the world's third largest producer and consumer of pork and pork products, and in recent years, it has been either the world's largest or second-largest exporter of pork and pork products [25]. Despite the potential economic consequences of an FAD outbreak in the US, there is a lack of published assessments identifying those areas of highest likelihood for FAD introduction [8].

Given the importance of the US livestock sector and the current distribution of ASF, CSF, and FMD, our objective was to outline a method to identify locations of relatively higher risk of FAD introduction into the US where surveillance measures could be more cost-effectively implemented.

Previous risk evaluations that identified a specific pathway of introduction specified legal and illegal importations of live animals, animal products, animal feed, genetic material, and bioterrorism as the most likely routes of FAD introduction [8]. These introduction pathways were reinforced by the U.S.-specific risk evaluations and align with the US Department of Agriculture's (USDA) analysis concluding that the illegal entry of swine products and byproducts presents the largest potential pathway for the entry of African swine fever virus (ASFV), and air passenger baggage and foreign mail are two of the largest illegal pathways [26]. It is important to note that since these risk assessments were produced, feed has been recognized as a possible pathway of introduction for ASF to the United States [27]. However, we have chosen for this study to exclude this introductory pathway from our analysis for all the reasons detailed in the Discussion section.

Using data representing these important potential introduction pathways, we applied a directional risk-ranking framework to identify areas with greatest likelihood of FAD introduction. We expect that our results will have utility in informing FAD surveillance and also in designing studies to better understand and characterize FAD risks.

## 2. Materials and Methods

To identify regions in the United States at higher risk for the potential introduction of FADs into domestic host populations, we employed a risk ranking approach for directional risks associated with initial disease introduction [14, 28]. Our risk ranking approach considers the relative risks associated with FAD introduction into an area, the host abundance in those areas, and the likelihood of infected material contaminating the local herds. Thus, we expect that the risk of FAD introduction is highest in locations where high density domestic host populations have frequent contact with high amounts of FAD contaminated products.

In spatial epidemiology, the choice of an appropriate geographical unit of analysis is a key decision that will influence most aspects of the study [29]. We chose to perform our analysis of the risk of introduction at the level of the Agricultural Statistics District [30] (ASD), a defined grouping of counties in each state by geography, climate, and crop practices. This unit choice represents a compromise between having a unit large enough to get reliable rates and not obscuring meaningful local variation [31, 32].

The simplest proxy for the risk of introduction of disease *d* (FMD, ASF, or CSF), ASD *a*, and species or set of species *l*, can be defined as the product of the quantity of potentially contaminated material by disease *Q*_*d*,*a*_ by the sum across all operation size classes *s* of the number of operations of livestock species *l* of that class size multiplied by the contamination risk for species *l* and class size *s*, divided by the area of ASD *a*

(1) $$\begin{array}{c} {IntroRisk}_{d,a,l=}Q_{d,a}*\frac{\sum_{s}\left({\#\text{⁣}operations}_{l,a,s}*\;{ContaminationRisk}_{l,s}\right)}{{area}_{a}}={QMI}_{d,a}*\;\underset{s}{\sum }{permeability}_{s,a}\text{.} \end{array}$$

ASF and CSF are swine-specific diseases, so only their introduction into domestic swine farms was considered. Given that cattle and domestic swine are the most commonly raised livestock species in the U.S. that are susceptible to FMD, each species was considered separately.

The first step was to create an index *Q*_*d*,*a*_ measuring the amount of potentially contaminated meat products entering each the ASDs, which we labelled quarantine material interceptions (QMI). To create this index, we used three data sources. The first source is the Agricultural Quarantine Inspection Monitoring (AQIM) air passenger inspection data, which represents a randomized sample of air passengers whose baggage is manually inspected for agricultural contraband. AQIM reports detailed information on the country of origin, airport of inspection, destination of the passengers, and the number and type of items intercepted. The second source is the AQIM USPS/Foreign Mail. It contains the same information as the air passenger pathway but is based on a random inspection of foreign mail packages arriving at USPS international mail facilities. The countries of origin and full mailing (destination) addresses are known. The last source is Mail287, the targeted USPS/foreign mail interception data. It is analogous to the AQIM USPS/Foreign Mail pathway, but these are interceptions from targeted inspections, unlike the AQIM databases.

We started by downloading the Topologically Integrated Geographic Encoding and Referencing (TIGER) county FIPS data and “places” databases (the places database is a geographic dataset of incorporated cities and unincorporated towns.), and compared the city and state destination data listed in our three data sources against the TIGER data. We used a string-matching algorithm with a maximum Jaro-Winkler string distance of 0.3 to return the closest match for a place name, and matches with high distances were evaluated for accuracy. Any record with a string distance of 0.1 or less was retained for further analysis. The geographic cleaning procedure was applied to all records for the years 2013–2020 (2,098,850 records). We dropped approximately 20% of the overall data due to place name misspellings, blank or unknown destination locations, and incorrect city/state pairings (e.g., Las Vegas, FL), for a total of 1,674,689 cleaned records.

A list of countries affected by ASF, CSF, and FMD was developed such that each country had a disease status by year, as well as a list of commodities potentially contaminated by each pathogen (see Supplementary Materials S1.1 and S1.2). To account for the recent detection of ASF in the Dominican Republic and Haiti (OIE-WAHIS (OIE-WAHIS. Available online: [33, 34] (accessed on November 11, 2021))), we added these countries as having a positive disease status for ASF. Both lists were used to filter records for interceptions of interest (defined as QMI for the commodity of interest coming from an affected country). The full dataset of air and mail interception dataset was evaluated for the years 2013-2020.

For each ASD, we computed the number of interceptions of interest (a commodity of interest intercepted in passenger baggage or from a mail package from a country of origin affected by a particular disease) for each year. Because the number of flights in 2020 was reduced due to COVID-19, the inspection counts for 2020 data were upscaled by county by performing a single draw from a Poisson using the mean of the inspection counts over the last three years. The number of interceptions for 2020 (without COVID-19) was then recalculated as the product of the new inspection count and the original ratio of interceptions to inspections for 2020. Finally, we computed the QMI index-a gauge of the amount of potentially contaminated material entering the ASD-by performing a single draw from a Poisson distribution using the mean of the interception count over the 2018–2020 (rescaled) period and by aggregating all resulting estimated interceptions at the ASD level. The resulting QMI values in 2020 were then used for the disease risk estimations.

The number of operations by species (swine, beef, or dairy) and size class (backyard, small, medium, or large) is from the National Agriculture Statistics Service (NASS) for the last available census year (2017). The size of an operation is a fair predictor of the breadth of the biosecurity measures implemented (food waste feeding; veterinary care; movements on and off the premises; contact with humans…), under the assumption that backyard operations do not have the same financial means or face the same degree of veterinary or regulatory supervision as large commercial entities (under some circumstances domestic swine operations may be under higher regulatory supervision if they are feeding waste). We thus posited that the percent of livestock operations of a given size and species that had at least one veterinarian visit in the previous year was a measure of their investment in animal health, so that the risk of contamination is inversely proportional to that number.

## 3. Results

Two ASDs (maps of ASDs by state can be found at [35]) in California (ASDs 40 and 80) were identified as high-risk recipients of potentially contaminated meat products for ASF (originating mostly from China and the Dominican Republic/Haiti), as well as ASDs in Florida (50), Oregon (10), Massachusetts (10), and Washington (10) (see Supplementary Material, S2). ASDs located in the states of Florida (50 and 80) and Connecticut (10) showed the highest QMIs for CSF (coming predominantly from Brazil). For FMD, potential spots were identified in California (80 and 40), followed by Washington (10), Oregon (10), Texas (90 and 40), and Arkansas (80). The most common origin for the products was China.

The percent of operations that had at least one veterinarian visit in the previous year–proxy for investment in animal health-was extracted from the most recent reports on livestock management practices released by USDA-APHIS for swine, dairy, and beef [36–38]. When a percentage was not available for a class size, the percentage or average for the nearest class size was used. Maps of the swine and cattle permeability indices (the second factor of formula (1) for each disease scheme involving swine and FMD in cattle) can be found in Supplementary Material S3. The highest swine permeability indices can be found in ASDs in Ohio, Pennsylvania, Maryland, Indiana, and Iowa. They are mostly driven by a large concentration of backyard operations (less than 25 pigs), except in Iowa, which has a high density of large operations (more than 1,000 animals). The largest cattle permeability indices are in Kentucky, Texas, Missouri, Tennessee, Arkansas, and Oklahoma. They are mostly driven by large concentrations of backyard beef operations (less than 10 animals).

**Figure 1:**
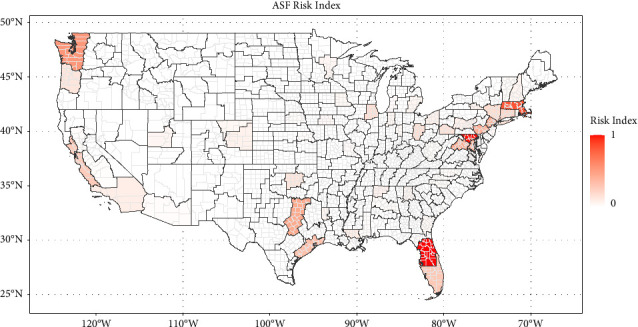
Map of the African swine fever risk index by an agricultural statistical district.

**Figure 2:**
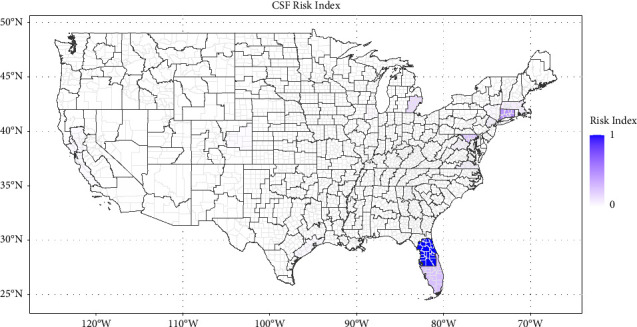
Map of the classical swine fever risk index by agricultural statistical district.

**Figure 3:**
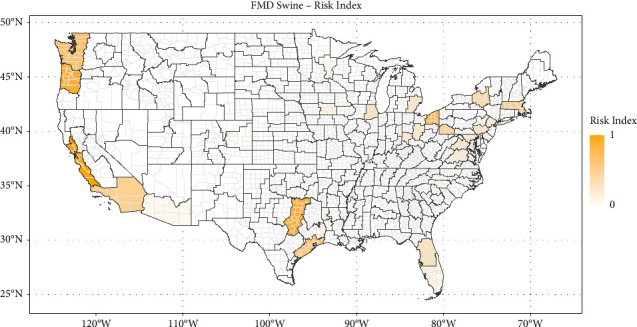
Map of the foot-and-mouth disease risk index for swine by agricultural statistical district.

**Figure 4:**
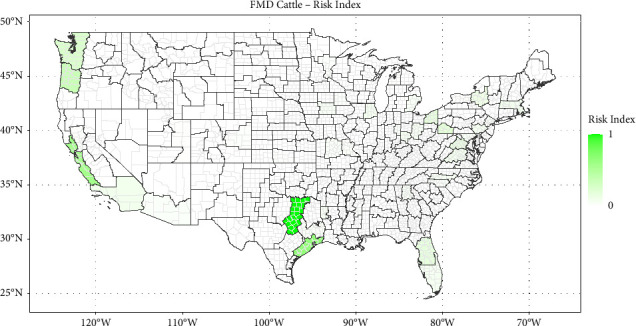
Map of foot-and-mouth disease risk index for cattle by agricultural statistical district.

Figures 1–4 show the heat maps of the risk indices for ASF (swine), CSF (swine), FMD (swine), and FMD (cattle), respectively. For an ASD to receive a large risk index, it needs to be both the recipient of a relatively important quantity of potentially contaminated meat products (high QMI index) and have a high density of livestock operations with lower biosecurity measures (high livestock permeability index). These maps can thus be interpreted as the overlay of the maps in Supplementary Materials S2 and S3. The three ASDs with the highest risk indices are presented in Table 1 for each disease scheme.

The ASDs with the highest risk of the introduction of African swine fever were identified as ASD 50 in Florida, encompassing 22 counties in central Florida, and ASD 20 in Maryland, which comprises eight counties in the north-central part of the state. ASD 50 in Florida is the third highest recipient of potentially contaminated meat products, originating mostly from Italy and the Dominican Republic/Haiti (QMI Index = 0.86); it has a midrange swine permeability index (0.50). Conversely, ASD 20 in Maryland has the third largest swine permeability index (0.92), driven by the relatively high density of backyard operations. The majority of the QMIs for this ASD stemmed from Ukraine, followed by the Philippines and China (QMI Index = 0.46). The following ASD by decreasing ASF risk index is ASD 10 in Massachusetts.

The ASD with the highest risk of the introduction of classical swine fever is ASD 50 in Florida, encompassing 22 counties in central Florida. It displays both a high swine permeability index (0.76) and the highest CSF QMI (1.00), originating almost exclusively from Brazil. The following two ASDs - Connecticut (10) and Florida (80) - exhibited a significantly lower risk index (0.46 and 0.27, respectively).

The ASD with the highest risk of the introduction of foot-and-mouth disease in swine is ASD 40 in California, comprising 13 counties along the Pacific Coast. It exhibits a low swine permeability index (0.17) but is the recipient of the highest amount of QMI for FMD, originating mostly from China. The following ASDs, 10 in Oregon and 40 in Texas, have comparable FMD risk indices (0.90 and 0.87, respectively).

The ASD with the highest risk of the introduction of foot-and-mouth disease in cattle is ASD 40 in Texas, comprising 27 counties in the northeastern part of the state. It is the recipient of a relatively low quantity of QMIs (index 0.17–originating mostly from China) but displays a high cattle permeability index (0.80), driven by the high density of backyard and small beef operations. ASDs 90 in Texas and 40 in California have lower FMD risk indices for cattle (0.65 and 0.57, respectively).

## 4. Discussion

We acknowledge that this method has potential limitations. The estimated detection rate of smuggled and improperly imported meat products is relatively low [10], which implies that the quantity of prohibited animal products potentially contaminated with the pathogens of interest entering the U.S. is estimated and thus subject to uncertainty. Additionally, because the list of countries affected by ASF, CSF, and FMD was established by WOAH on a per-country basis, we may overestimate the risk of the presence of a disease when the disease is circumscribed to a specific region within a country. As an example, over the study period, the presence of CSF was restricted to the northern part of Brazil [20], but the whole country is listed as a potential origin for products contaminated with CSF. Moreover, some states allow swine operations to feed their pigs human food waste that contains or has had contact with meat, poultry, or fish. This practice is otherwise referred to as swill or garbage feeding. The Swine Health Protection Act allows each state to determine whether garbage feeding is allowed within their state. In a state where it is allowed, owners must be licensed, and this food waste must be properly handled and cooked. If these requirements are not fully met, this practice can spread diseases if contaminated meat products are fed to pigs. The increased risk of contamination is difficult to quantify and has therefore not been accounted for in our risk ranking of agricultural districts. Finally, the mechanism of transmission from contaminated meat products to livestock operations is not fully understood. In particular, our analysis did not specifically include the potential for wildlife to be involved in the introduction of these diseases to the US. Wild pigs are the most likely species to potentially be involved in an outbreak of these diseases [12, 39]. Wild pigs have invaded the majority of U.S. states, and previous work suggests that they could play an important role in the maintenance and transmission of an FAD introduction [8, 17].

On a further note, it has been shown under experimental conditions that ASFV can survive when inoculated into a particular feed or feed components [40]. Reference [40] inoculated various animal feed ingredients with viral pathogens, including ASFV, to test their viability under transboundary shipping conditions and found that conventional soybean meal, organic soybean meal, soy oil cake, choline, moist cat food, moist dog food, dry dog food, and pork sausage casings all have an environmental matrix capable of supporting ASFV stability. Significant quantities of these products, including soybean products, which are used as a source of protein in swine diets, are imported into the United States from around the world every year and may serve as a pathway for introducing and transmitting ASFV [41]. However, there is substantial uncertainty and variability in recent transoceanic models assessing the probability of ASF reaching the United States via this pathway. The median probability of one vessel with ASFV-contaminated product entering the United States was estimated to be once every 1563−21 years for soybeans (based on the recontamination assumption) and once every 50 years for corn [42]. It is difficult to determine what have historically been the exact pathways of introduction for ASFV into nonendemic regions. However, these pathways can be broken down into either local transmissions or long-distance jumps. The most recent ASF epidemics in Central and Eastern Europe are thought to be perpetuated by a wild boar-habitat epidemiological cycle [43]. Many first reports of ASFV are often in feral swine. For instance, Bulgaria reported ASF in a wild boar on October 23, 2018 along its northern border with Poland, another ASFV-positive country, months before the first domestic outbreak in August [44]. As of January 20, 2023, along Greece's border with southern Bulgaria, a case of ASFV was once again detected in a wild boar [45]. In cases where the virus has spread between countries without borders or island countries (i.e., a long-distance jump), human activity such as trade and travel is the suspected cause [46]. For instance, a 2007 outbreak of ASFV in Georgia was theorized to be from a ship near the Black Sea Port of Poti due to the region where the first case was observed [47]. Furthermore, ASFV from domestic pigs in the 2007 Georgia outbreak was isolated, analyzed, and found to be closely related to those circulating in Mozambique, Madagascar, and Zambia, which further supports the long-distance transmission of this virus [48]. It is believed that contaminated pork products brought in on ships are what introduced ASFV to Georgia, but there are different theories as to the exact mechanism of transmission [46, 48]. Both the improper disposal of contaminated pork meat from a docked ship and the feeding of contaminated meat products directly to pigs have been theorized [46, 48]. Georgia is one of the few documented cases of long-distance transmission pathways that can provide traceback and theorize the origin of the introduction. Of the cases that have been reported, feed or feed components have not been theorized as the mechanism for introduction to a nonendemic region. Finally, although we have an idea of what ports many high-risk imports arrive at [49], we cannot trace these goods as they move throughout the United States. The North American Industry Classification System (NAICS) is used to categorically group industries into a hierarchical structure to collect, analyze, and publish statistical data related to the United States business economy [50]. This system is used by the International Trade Administration database to visualize global markets and the movement of goods into and throughout the United States. However, the International Trade Administration database only gets as specific as the NAICS-4 code, which is only as detailed as the industry group level [47, 50]. This makes it difficult to trace specific feeds of interest from ASFV-positive countries. As a conclusion, while a theoretical risk exists, there do not appear to be any documented reports of an outbreak being traced back to feed or feed components at this time. Furthermore, the data are not available to accurately trace the movement of feedstuffs throughout the United States once they arrived in the country. Hence, for this paper, we have chosen to exclude this introductory pathway for all the aforementioned reasons and acknowledge the limitation this places on our analysis.

## 5. Conclusions

Given the global interconnectedness and the number of countries endemic with ASF, CSF, and FMD, the risk of introduction of the one of these FADs into the U.S. is of concern, prompting surveillance programs to mitigate the risks [14, 51]. Once introduced, the cost of disease management and control for any of these diseases is indeed estimated to be in the billions of dollars [52]. Preparedness is crucial to successful prevention, control, and eradication of any one of these diseases, and preventing large economic losses is contingent on our ability to rapidly and accurately detect an outbreak and protect vulnerable elements of the system [6, 53].

**Figure 5:**
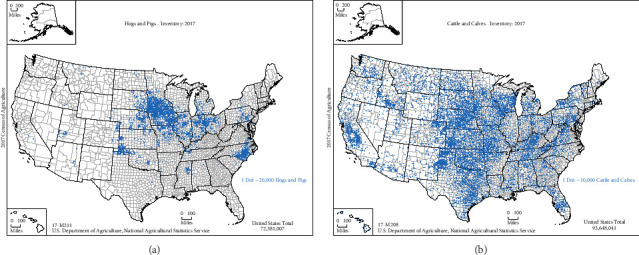
Inventory of hogs, pigs, cattle, and calves (2017) [54].

Locations with the highest introduction risks for all diseases in swine were identified in areas with relatively small concentrations of industry. Introduction risk is estimated to be highest in Florida for ASF and CSF, and California for FMD in swine (see Figures 1–3) while U.S. hog operations tend to be heavily concentrated in the Midwest—Iowa and southern Minnesota, particularly—and in eastern North Carolina, but also in Oklahoma and Texas (see Figure 5). In the same way, as the risk of introduction of FMD in cattle is highest in an ASD in Texas (see Figure 4), the beef industry has a myriad of small operators, especially among the cow/calf sectors (see Figure 5).

This disconnection between introduction risk and livestock industry presence should somewhat be protective in terms of buffering domestic economic impacts. But an incursion of any of those viruses could have sizeable trade implications. While the fundamental animal health issues create problems for producers, a major driver of the economic impacts are the trade disruptions from disease-related import restrictions. International meat markets have been increasingly affected by animal disease outbreaks, which have caused trade diversion and shifted market shares between exporters of the same and different types of meat products [23].

Since January 2021, seven countries have reported ASF as a first occurrence in their country, while nine countries have reported its spread to new zones [19]. While the permeability index defined in (1) is likely stable over a few years, the quantity of potentially contaminated material by disease in a given county could fluctuate to some extent over the same period. The results could thus be available updated when the AQIM, the AQIM USPS/Foreign Mail, and the Mail287 datasets used for the study become available. Leveraging the most up-to-date information on the most likely places of introduction, future work should focus on understanding the potential relative economic impact of ASF, CSF, and FMD on the U.S. economy using disease simulation models to predict their spread and an economic models to determine the relative economic impacts on U.S. domestic and export markets. The consequences of these most likely scenarios should also be contrasted against the worst-case scenarios for each disease.

## Figures and Tables

**Table 1:** Three ASDs with the highest risk index by disease/species scheme.

Disease/species	ASD #1	Risk index	ASD #2	Risk index	ASD #3	Risk index
ASF/swine	Florida (50)	1.00	Maryland (10)	0.99	Massachusetts (10)	0.82
CSF/swine	Florida (50)	1.00	Connecticut (10)	0.46	Florida (80)	0.27
FMD/swine	California (40)	1.00	Oregon (10)	0.90	Texas (40)	0.87
FMD/cattle	Texas (40)	1.00	Texas (90)	0.65	California (40)	0.57

## Data Availability

The data used to support the findings of this study were supplied by Customs and Border Protection under an MOU and so cannot be made freely available. Requests for access to these data should be made to Customs and Border Protection.

## Supplementary Materials

Supplementary Materials S1: list of countries by disease status and potentially contaminated commodities. Table S1.1 lists for each country (column 1) and year (column 2) the diseases that have been reported to the World Animal Health Organization. Table S1.2 lists for each commodity (column 1) the potential presence of ASF or CSF (column 2) and FMD (column 3). S2: maps of QMI. Three maps present an index ranging from 0 to 1 of the amount of potentially contaminated meat products (ASF, CSF, and FMD) by agricultural state district, with 0 being the lowest risk and 1 being the highest. S3: maps of livestock permeability indices. Two maps present an index ranging from 0 to 1 of the permeability of the swine and cattle livestock industries (defined in equation 1) by agricultural state district, with 0 being the lowest risk and 1 being the highest.

## References

[1] Rossiter P. B., Al Hammadi N. (2009). Living with transboundary animal diseases (TADs). *Tropical Animal Health and Production*.

[2] Fao (2019). Prevention System (EMPRES) TADs Agriculture and Consumer Protection Department. *Animal Production and Health*.

[3] Arzt J., White W. R., Thomsen B. V., Brown C. C. (2010). Agricultural diseases on the move early in the third millennium. *Veterinary pathology*.

[4] Schlundt J., Kock R. A., Fisher J. (2002). Infectious animal diseases: the wildlife/livestock interface. *Revue Scientifique et Technique de l'OIE*.

[5] Plowright R. K., Reaser J. K., Locke H. (2021). Land use-induced spillover: a call to action to safeguard environmental, animal, and human health. *The Lancet Planetary Health*.

[6] Gregorio D. I., DeChello L. M., Samociuk H. (2013). Proposed terms and concepts for describing and evaluating animal-health surveillance systems. *Preventive Veterinary Medicine*.

[7] Fanelli A., Muñoz O., Mantegazza L., De Nardi M., Capua I. (2022). Is the COVID‐19 pandemic impacting on the risk of African Swine Fever virus (ASFV) introduction into the United States? A short‐term assessment of the risk factors. *Transboundary and Emerging Diseases*.

[8] Brown V. R., Miller R. S., McKee S. C. (2021). Risks of introduction and economic consequences associated with African swine fever, classical swine fever and foot‐and‐mouth disease: a review of the literature. *Transboundary and emerging diseases*.

[9] Niederwerder M. C. (2021). Risk and mitigation of African swine fever virus in feed. *Animals*.

[10] Jurado C., Mur L., Pérez Aguirreburualde M. S. (2019). Risk of African swine fever virus introduction into the United States through smuggling of pork in air passenger luggage. *Scientific Reports*.

[11] Wormington J. D., Golnar A. J., Poh K. C. (2019). Risk of african swine fever virus sylvatic establishment and spillover to domestic swine in the United States. *Vector Borne and Zoonotic Diseases*.

[12] Brown V. R., Bevins S. N. (2018). A review of african swine fever and the potential for introduction into the United States and the possibility of subsequent establishment in feral swine and native ticks. *Frontiers in Veterinary Science*.

[13] Herrera-Ibatá D. M., Martínez-López B., Quijada D., Burton K. R., Mur L. (2017). Quantitative approach for the risk assessment of African swine fever and Classical swine fever introduction into the United States through legal imports of pigs and swine products. *PLoS One*.

[14] Miller R. S., Bevins S. N., Cook G. (2022). Adaptive risk‐based targeted surveillance for foreign animal diseases at the wildlife‐livestock interface. *Transboundary and Emerging Diseases*.

[15] Pritchett J. G., Thilmany D. D., Johnson K. K. (2005). Animal disease economic impacts: a survey of literature and typology of research approaches. *The International Food and Agribusiness Management Review*.

[16] Blayney D. P., Dyck J. H., Harvey D. J. (2006). Economic effects of animal diseases linked to trade dependency. *Amber Waves*.

[17] Miller R. S., Sweeney S. J., Slootmaker C. (2017). Cross-species transmission potential between wild pigs, livestock, poultry, wildlife, and humans: implications for disease risk management in North America. *Scientific Reports*.

[18] McElwain T. F., Thumbi S. M. (2017). Animal pathogens and their impact on animal health, the economy, food security, food safety and public health. *Revue Scientifique et Technique de l'OIE*.

[19] World Organisation for Animal Health (2022). African swine fever. https://www.woah.org/en/disease/african-swine-fever/.

[20] Urbano A. C., Ferreira F. (2022). African swine fever control and prevention: an update on vaccine development. *Emerging Microbes and Infections*.

[21] World Organisation for Animal Health (2022). Classical swine fever. https://www.woah.org/en/disease/classical-swine-fever/.

[22] World Organisation for Animal Health (2022). Foot-and-mouth disease. https://www.woah.org/en/disease/foot-and-mouth-disease/.

[23] Morgan N., Prakash A., Jutzi S. (2006). Influencia de las enfermedades animales en los mercados agropecuarios internacionales: -EN- International livestock markets and the impact of animal disease -FR- lmpact des maladies animales sur les échanges internationaux d’animaux d’élevage et de leurs produits -ES-. *Revue Scientifique et Technique de l'OIE*.

[24] Economic Research Service (2022). Animal products. https://www.ers.usda.gov/topics/animal-products/.

[25] Economic Research Service (2022). Hogs and pork. https://www.ers.usda.gov/topics/animal-products/hogs-pork/.

[26] Usda Aphis V. S. (2021). Qualitative assessment of the likelihood of african swine fever virus entry to the united states: entry assessment.” usda: aphis: vs: center for epidemiology and animal health, risk assessment team. https://www.aphis.usda.gov/animal_health/downloads/animal_diseases/swine/asf-entry.pdf.

[27] Shurson G. C., Palowski A., Ligt J. L. G. (2022). New perspectives for evaluating relative risks of African swine fever virus contamination in global feed ingredient supply chains. *Transboundary and Emerging Diseases*.

[28] Miller R. S., Pepin K. M. (2019). Board invited review: prospects for improving management of animal disease introductions using disease-dynamic models. *Journal of Animal Science*.

[29] Arsenault J., Michel P., Berke O., Ravel A., Gosselin P. (2013). How to choose geographical units in ecological studies: proposal and application to campylobacteriosis. *Spatial and spatio-temporal epidemiology*.

[30] https://www.nass.usda.gov/Data_and_Statistics/County_Data_Files/Frequently_Asked_Questions/index.php.

[31] Gregorio D. I., DeChello L. M., Samociuk H., Kulldorff M. (2005). Lumping or splitting: seeking the preferred areal unit for health geography studies. *International Journal of Health Geographics*.

[32] Osypuk T. L., Galea S. (2007). What level macro? Choosing appropriate levels to assess how place influences population health. *Macrosocial Determinants of Population Health*.

[33] https://wahis.oie.int/#/report-info?reportId=36844.

[34] https://wahis.oie.int/#/report-info?reportId=39928.

[35] https://www.nass.usda.gov/Charts_and_Maps/Crops_County/boundary_maps/indexgif.php.

[36] USDA (2009). *Beef 2007-08, Part II: Reference of Beef Cow-Calf Management Practices in the United States, 2007-08*.

[37] USDA (2015). *Swine 2012 Part I: Baseline Reference of Swine Health and Management in the United States, 2012”*.

[38] USDA (2016). *Dairy 2014, “Dairy Cattle Management Practices in the United States, 2014” USDA–APHIS–VS–CEAH–NAHMS*.

[39] Brown V. R., Bevins S. N. (2018). A review of African swine fever and the potential for introduction into the United States and the possibility of subsequent establishment in feral swine and native ticks. *Frontiers in Veterinary Science*.

[40] Dee S. A., Bauermann F. V., Niederwerder M. C. (2019). Correction: survival of viral pathogens in animal feed ingredients under transboundary shipping models. *PLoS One*.

[41] Niederwerder M. C. (2021). Risk and mitigation of african swine fever virus in feed. *Animals*.

[42] Schambow R. A., Sampedro F., Urriola P. E., Ligt J. L. G., Perez A., Shurson G. C. (2022). Rethinking the uncertainty of African swine fever virus contamination in feed ingredients and risk of introduction into the United States. *Transboundary and Emerging Diseases*.

[43] Chenais E., Depner K., Guberti V., Dietze K., Viltrop A., Ståhl K. (2019). Epidemiological considerations on African swine fever in Europe 2014-2018. *Porcine Health Management*.

[44] Zani L., Dietze K., Dimova Z. (2019). African swine fever in a Bulgarian backyard farm-a case report. *Veterinary Sciences*.

[45] National Disease Control Centre (2023). African swine fever update (vol. 1). https://assets.gov.ie/245394/8c65615b-2b3d-4eed-8aed-7f7b217d2ee8.pdf.

[46] Jiang D., Ma T., Hao M. (2022). Quantifying risk factors and potential geographic extent of African swine fever across the world. *PLoS One*.

[47] International (2022). Tradestats express - trade by partner by state. https://www.trade.gov/data-visualization/tradestats-express-trade-partner-stat39.

[48] Rowlands R. J., Michaud V., Heath L. (2008). African swine fever virus isolate, Georgia, 2007. *Emerging Infectious Diseases*.

[49] Patterson G. (2022). An analysis of select swine feed ingredients and pork products imported into the United States from African swine fever virus affected countries. *Transboundary and Emerging Diseases*.

[50] United States Census Bureau (2022). Naics codes and understanding industry classification systems. https://www.census.gov/programs-surveys/economic-census/year/2022/guidance/understanding-naics.html.

[51] USDA (2019). *Swine Hemorrhagic Fevers: African and Classical Swine Fever Integrated Surveillance Plan*.

[52] Schroeder T. C., Pendell D. L., Sanderson M. W., Mcreynolds S. (2015). Economic impact of alternative FMD emergency vaccination strategies in the midwestern United States. *Journal of Agricultural and Applied Economics*.

[53] Bajardi P., Barrat A., Savini L., Colizza V. (2012). Optimizing surveillance for livestock disease spreading through animal movements. *Journal of The Royal Society Interface*.

[54] Nass (2017). Census ag. atlas maps. https://www.nass.usda.gov/Publications/AgCensus/2017/Online_Resources/Ag_Atlas_Maps/.

