# Supplementary material for: Areas within the United States at the Highest Risk for African Swine Fever, Classical Swine Fever, and Foot-and-Mouth Disease Introduction
Source: Transbound Emerg Dis. 2023 May 25;2023:8892037. doi: 10.1155/2023/8892037 (PMC12016723; doi:10.1155/2023/8892037)

**Supplementary Materials**

S1: List of countries by disease status and potentially contaminated commodities

S2: Maps of QMI

S3: Maps of livestock permeability indices

**S1: List of countries with disease status and potentially contaminated commodities**

S1.1 List of countries by disease status

| Country | Year | Disease |
| --- | --- | --- |
| ITA | 2009 | ASF |
| LTU | 2009 | CSF |
| ISR | 2009 | CSF |
| MEX | 2009 | CSF |
| RUS | 2009 | CSF |
| AGO | 2009 | FMD |
| BHR | 2009 | FMD |
| CHN | 2009 | FMD |
| TWN | 2009 | FMD |
| COL | 2009 | FMD |
| EGY | 2009 | FMD |
| KWT | 2009 | FMD |
| ZAF | 2009 | FMD |
| ITA | 2010 | ASF |
| CAF | 2010 | ASF |
| TZA | 2010 | ASF |
| GTM | 2010 | CSF |
| BWA | 2010 | FMD |
| CHN | 2010 | FMD |
| KOR | 2010 | FMD |
| MNG | 2010 | FMD |
| MMR | 2010 | FMD |
| RUS | 2010 | FMD |
| ZWE | 2010 | FMD |
| ITA | 2011 | ASF |
| KEN | 2011 | ASF |
| LTU | 2011 | CSF |
| BGR | 2011 | FMD |
| BWA | 2011 | FMD |
| TWN | 2011 | FMD |
| ISR | 2011 | FMD |
| PRK | 2011 | FMD |
| LBY | 2011 | FMD |
| NAM | 2011 | FMD |
| ITA | 2012 | ASF |
| LVA | 2012 | CSF |
| GTM | 2012 | CSF |
| BWA | 2012 | FMD |
| TWN | 2012 | FMD |
| KAZ | 2012 | FMD |
| LBY | 2012 | FMD |
| PRY | 2012 | FMD |
| RUS | 2012 | FMD |
| ZAF | 2012 | FMD |
| ZMB | 2012 | FMD |
| ITA | 2013 | ASF |
| BEN | 2013 | ASF |
| TUR | 2013 | FMD |
| CHN | 2013 | FMD |
| MNG | 2013 | FMD |
| NAM | 2013 | FMD |
| RUS | 2013 | FMD |
| EST | 2014 | ASF |
| ITA | 2014 | ASF |
| LVA | 2014 | ASF |
| LTU | 2014 | ASF |
| POL | 2014 | ASF |
| UKR | 2014 | ASF |
| LVA | 2014 | CSF |
| MNG | 2014 | CSF |
| RUS | 2014 | CSF |
| TUR | 2014 | FMD |
| CHN | 2014 | FMD |
| GIN | 2014 | FMD |
| KOR | 2014 | FMD |
| MOZ | 2014 | FMD |
| NAM | 2014 | FMD |
| EST | 2015 | ASF |
| ITA | 2015 | ASF |
| LVA | 2015 | ASF |
| LTU | 2015 | ASF |
| POL | 2015 | ASF |
| UKR | 2015 | ASF |
| MNG | 2015 | CSF |
| UKR | 2015 | CSF |
| TUR | 2015 | FMD |
| DZA | 2015 | FMD |
| BWA | 2015 | FMD |
| TWN | 2015 | FMD |
| MRT | 2015 | FMD |
| MNG | 2015 | FMD |
| MAR | 2015 | FMD |
| MOZ | 2015 | FMD |
| ZAF | 2015 | FMD |
| ZWE | 2015 | FMD |
| EST | 2016 | ASF |
| ITA | 2016 | ASF |
| LVA | 2016 | ASF |
| LTU | 2016 | ASF |
| POL | 2016 | ASF |
| BDI | 2016 | ASF |
| KEN | 2016 | ASF |
| MDA | 2016 | ASF |
| ZAF | 2016 | ASF |
| UKR | 2016 | ASF |
| KOR | 2016 | CSF |
| RUS | 2016 | CSF |
| TUR | 2016 | FMD |
| AGO | 2016 | FMD |
| GNB | 2016 | FMD |
| IRN | 2016 | FMD |
| KOR | 2016 | FMD |
| KWT | 2016 | FMD |
| CZE | 2017 | ASF |
| EST | 2017 | ASF |
| ITA | 2017 | ASF |
| LVA | 2017 | ASF |
| LTU | 2017 | ASF |
| POL | 2017 | ASF |
| UKR | 2017 | ASF |
| CIV | 2017 | ASF |
| MDA | 2017 | ASF |
| RUS | 2017 | ASF |
| RUS | 2017 | CSF |
| TUR | 2017 | FMD |
| DZA | 2017 | FMD |
| CHN | 2017 | FMD |
| COD | 2017 | FMD |
| ISR | 2017 | FMD |
| KOR | 2017 | FMD |
| MWI | 2017 | FMD |
| RUS | 2017 | FMD |
| ZAF | 2017 | FMD |
| BEL | 2018 | ASF |
| BGR | 2018 | ASF |
| CZE | 2018 | ASF |
| EST | 2018 | ASF |
| HUN | 2018 | ASF |
| ITA | 2018 | ASF |
| LVA | 2018 | ASF |
| LTU | 2018 | ASF |
| POL | 2018 | ASF |
| ROU | 2018 | ASF |
| UKR | 2018 | ASF |
| CHN | 2018 | ASF |
| MDA | 2018 | ASF |
| BRA | 2018 | CSF |
| JPN | 2018 | CSF |
| RUS | 2018 | CSF |
| TUR | 2018 | FMD |
| CHN | 2018 | FMD |
| COL | 2018 | FMD |
| ISR | 2018 | FMD |
| KEN | 2018 | FMD |
| MWI | 2018 | FMD |
| MOZ | 2018 | FMD |
| NPL | 2018 | FMD |
| RUS | 2018 | FMD |
| SLE | 2018 | FMD |
| ZMB | 2018 | FMD |
| ZWE | 2018 | FMD |
| BEL | 2019 | ASF |
| BGR | 2019 | ASF |
| EST | 2019 | ASF |
| HUN | 2019 | ASF |
| ITA | 2019 | ASF |
| LVA | 2019 | ASF |
| LTU | 2019 | ASF |
| POL | 2019 | ASF |
| ROU | 2019 | ASF |
| SVK | 2019 | ASF |
| UKR | 2019 | ASF |
| KHM | 2019 | ASF |
| CHN | 2019 | ASF |
| CIV | 2019 | ASF |
| HKG | 2019 | ASF |
| KEN | 2019 | ASF |
| LAO | 2019 | ASF |
| MDA | 2019 | ASF |
| PHL | 2019 | ASF |
| RUS | 2019 | ASF |
| SRB | 2019 | ASF |
| ZAF | 2019 | ASF |
| TUR | 2019 | FMD |
| ISR | 2019 | FMD |
| MAR | 2019 | FMD |
| MMR | 2019 | FMD |
| PSE | 2019 | FMD |
| RUS | 2019 | FMD |
| ZAF | 2019 | FMD |
| BGR | 2020 | ASF |
| CHN | 2020 | ASF |
| DEU | 2020 | ASF |
| DOM | 2020 | ASF |
| HTI | 2020 | ASF |
| GRC | 2020 | ASF |
| LAO | 2020 | ASF |
| LVA | 2020 | ASF |
| MDA | 2020 | ASF |
| MMR | 2020 | ASF |
| NAM | 2020 | ASF |
| PHL | 2020 | ASF |
| POL | 2020 | ASF |
| SLE | 2020 | ASF |
| ZAF | 2020 | ASF |
| UKR | 2020 | ASF |
| ZMB | 2020 | ASF |
| BRA | 2020 | CSF |
| CHN | 2020 | FMD |
| MWI | 2020 | FMD |
| NAM | 2020 | FMD |
| RWA | 2020 | FMD |
| ZAF | 2020 | FMD |
| ZWE | 2020 | FMD |

S1.2 List of potentially contaminated commodities

| **Item** | **ASF/CSF** | **FMD** |
| --- | --- | --- |
| Animal Blood | X | X |
| Animal Food/Feed | X | X |
| Animal Gelatin | X | X |
| Animal Horn/Antler |  | X |
| Animal Part | X | X |
| Animal Skin | X | X |
| Animal Skull |  | X |
| Animal Trophy | X | X |
| Animal, Carcass | X | X |
| Animal, Live |  | X |
| Beef |  | X |
| Beef, Chorizo |  | X |
| Beef, Dry |  | X |
| Beef, Fat/Lard |  | X |
| Beef, Flake |  | X |
| Beef, Jerky |  | X |
| Beef, Processed |  | X |
| Beef, Sausage |  | X |
| Beef, Shredded |  | X |
| Cheese, Goat |  | X |
| Goat, Meat |  | X |
| Lamb |  | X |
| Lamb, Processed |  | X |
| Manure |  | X |
| Meat | X | X |
| Meat, Bologna |  | X |
| Meat, Broth |  | X |
| Meat, Canned |  | X |
| Meat, Cooked | X | X |
| Meat, Fat/Lard | X | X |
| Meat, Hamburger |  | X |
| Meat, Hot Dog | X | X |
| Meat, Minced | X | X |
| Meat, Preserved | X | X |
| Meat, Processed | X | X |
| Meat, Sandwich | X | X |
| Meat, Tamale | X | X |
| Meat, Wild Animal | X | X |
| Milk |  | X |
| Milk, Sweeten |  | X |
| Mooncake With Meat | X |  |
| Pepperoni | X | X |
| Pizza w/ Meat |  | X |
| Pork | X | X |
| Pork, Bacon | X | X |
| Pork, Chorizo | X | X |
| Pork, Dried | X | X |
| Pork, Fat/Lard | X | X |
| Pork, Ham | X | X |
| Pork, Processed | X | X |
| Pork, Salami | X | X |
| Pork, Sausage | X | X |
| Pork, Skin | X | X |
| Pudding, Blood | X | X |
| Rawhide |  | X |
| Sandwich, Meat | X | X |
| Soup, Mix w/ Meat | X | X |

**S2: Maps of QMI**


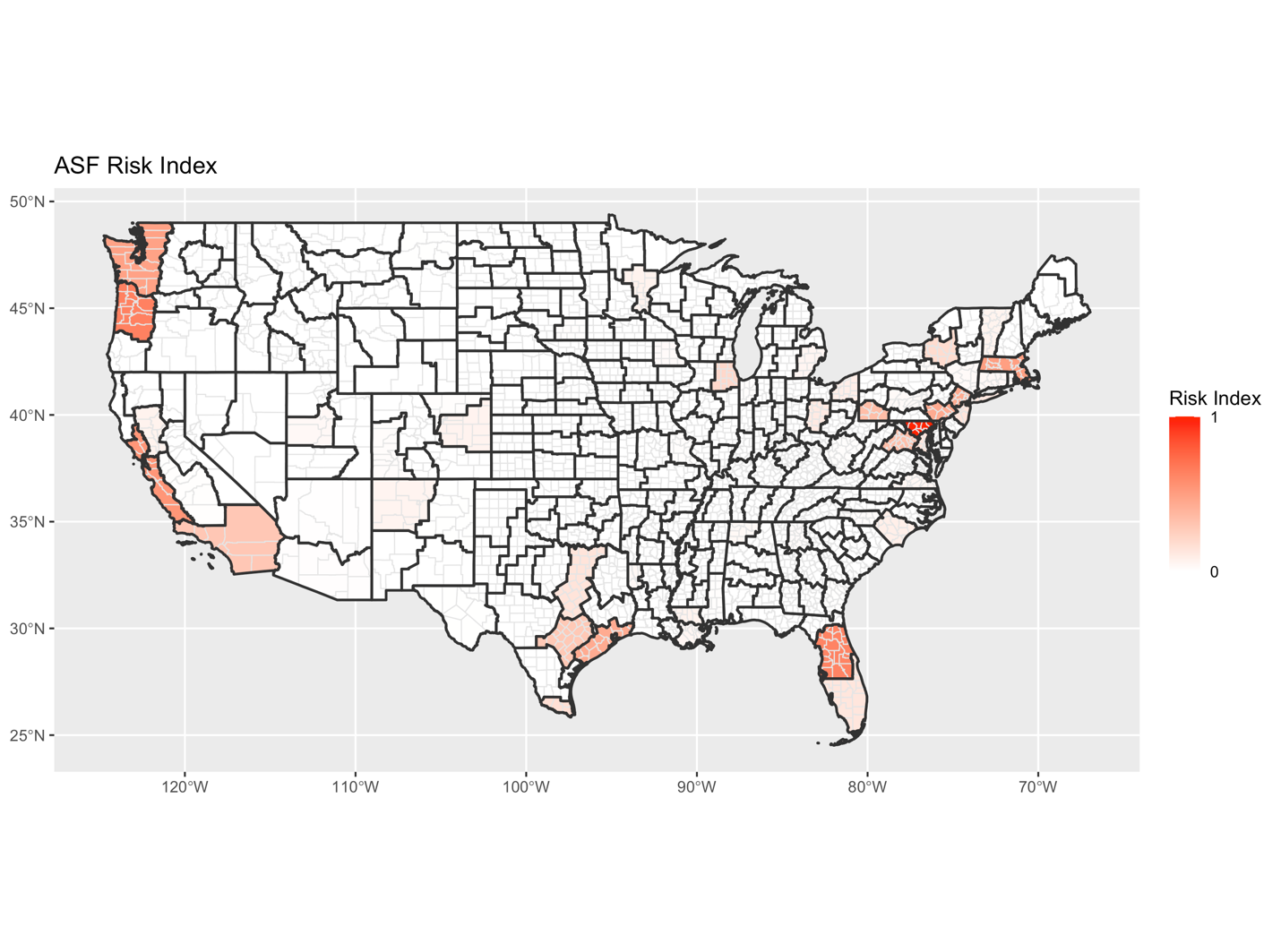


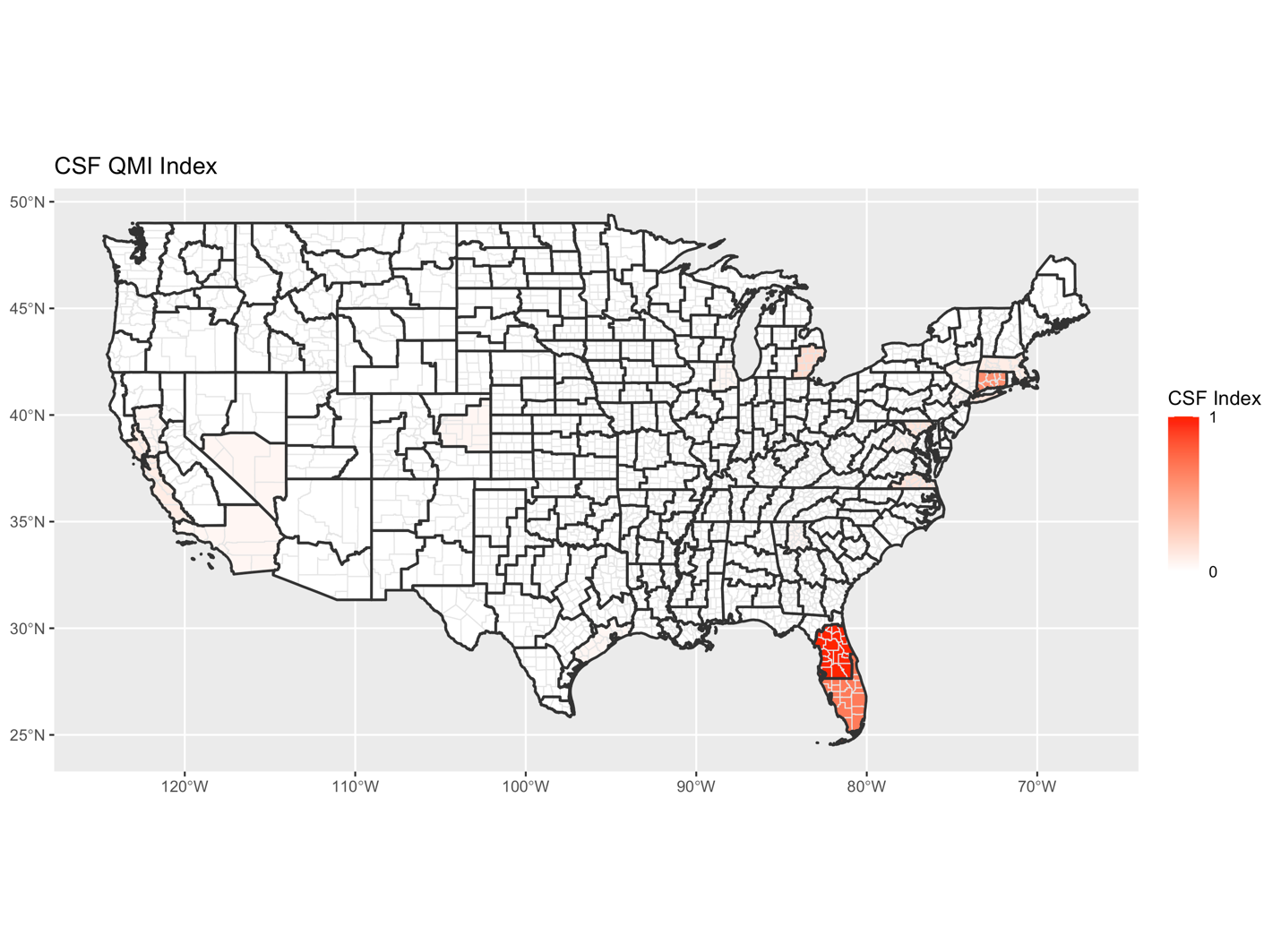


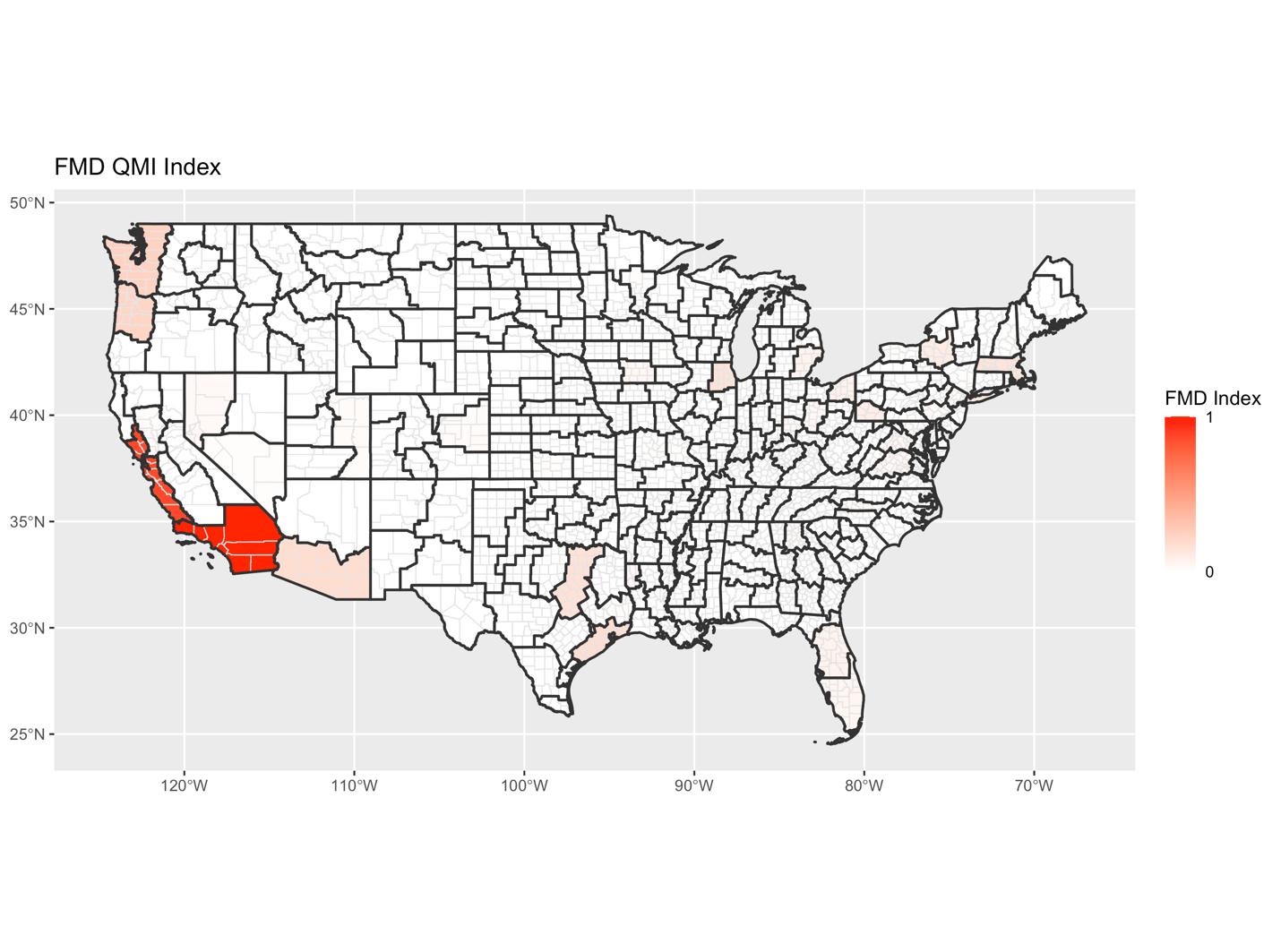


**S3: Maps of livestock permeability indices**


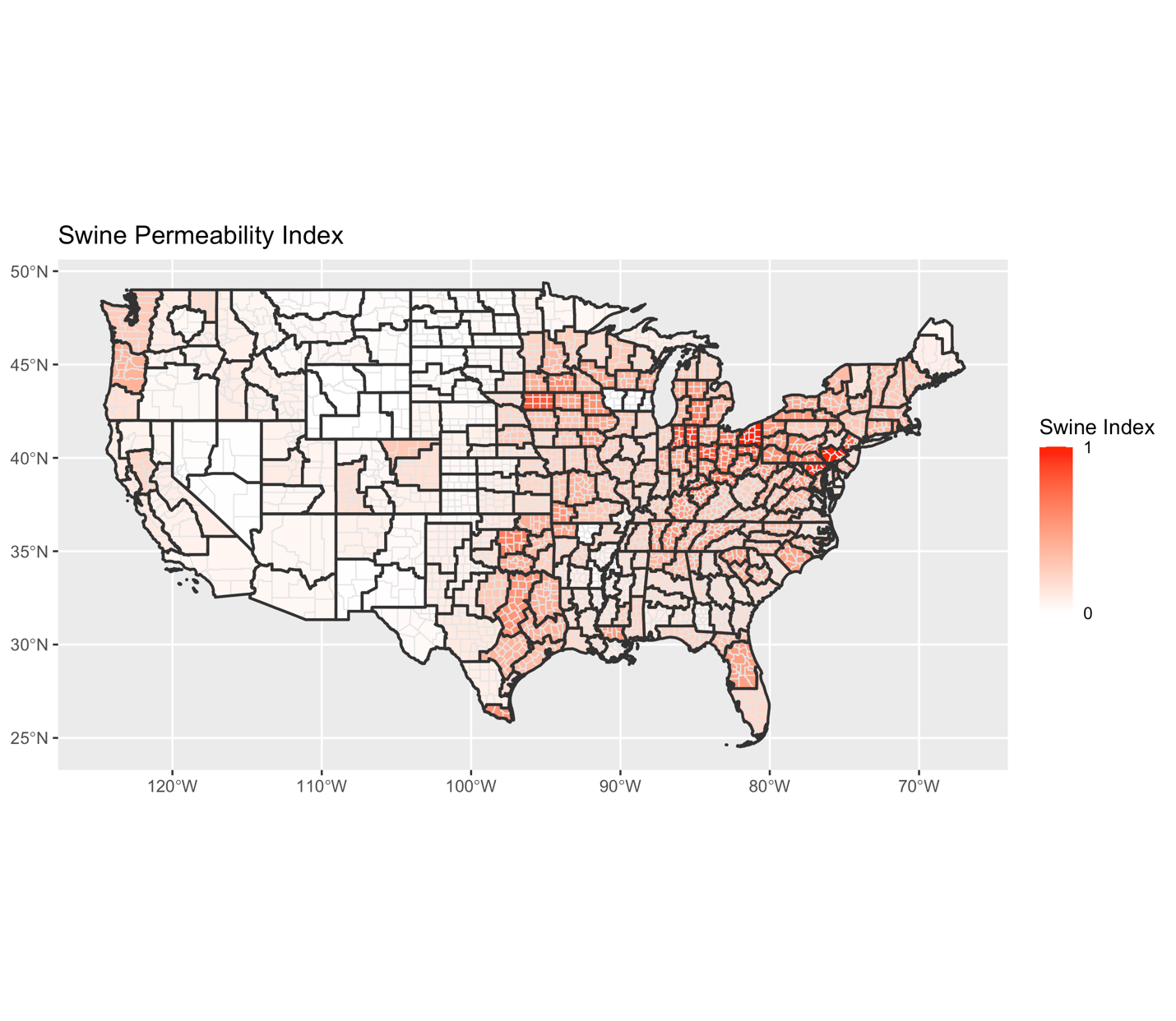


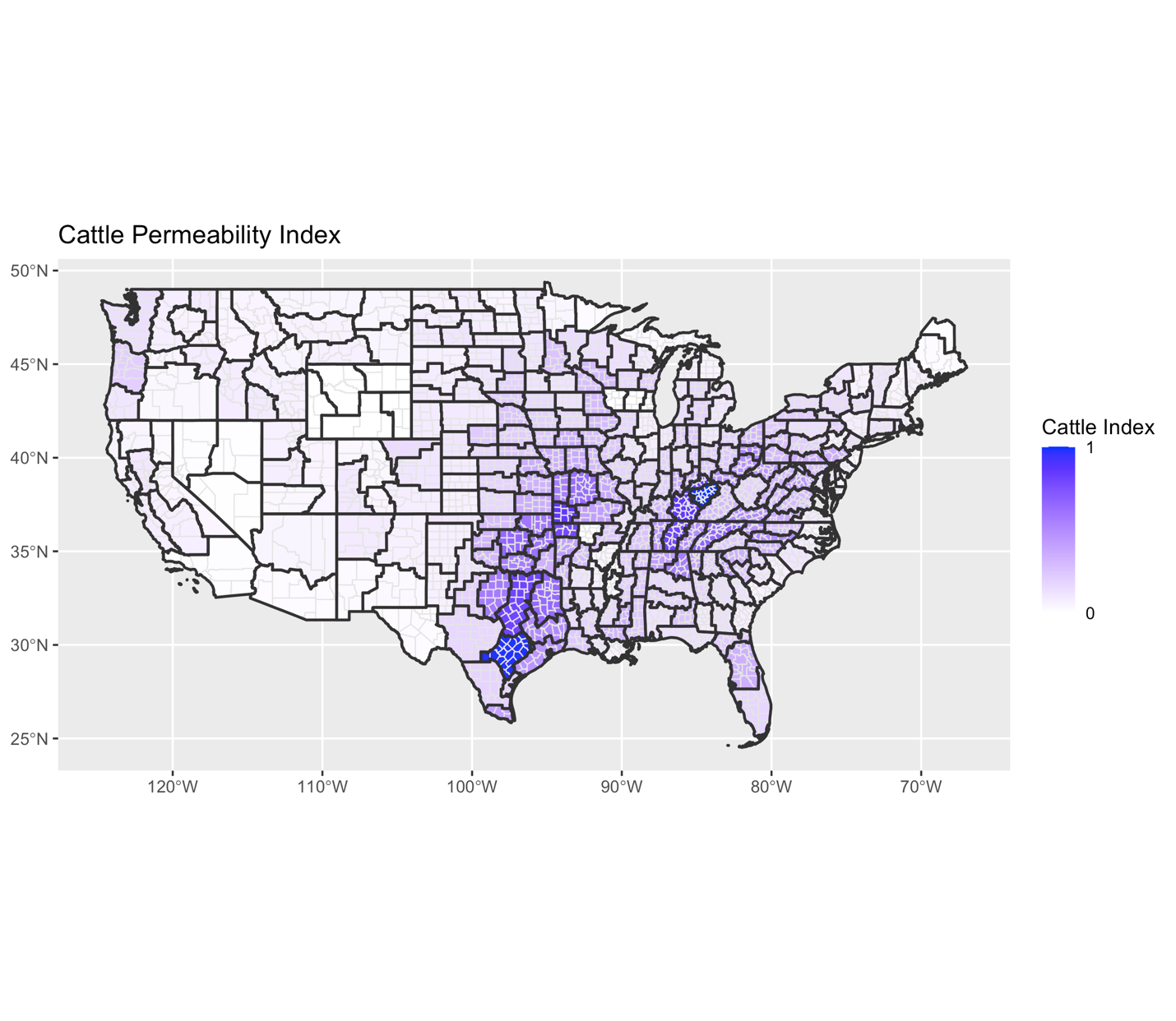

Supplement: Supplementary Materials — S1: list of countries by disease status and potentially contaminated commodities. Table S1.1 lists for each country (column 1) and year (column 2) the diseases that have been reported to the World Animal Health Organization. Table S1.2 lists for each commodity (column 1) the potential presence of ASF or CSF (column 2) and FMD (column 3). S2: maps of QMI. Three maps present an index ranging from 0 to 1 of the amount of potentially contaminated meat products (ASF, CSF, and FMD) by agricultural state district, with 0 being the lowest risk and 1 being the highest. S3: maps of livestock permeability indices. Two maps present an index ranging from 0 to 1 of the permeability of the swine and cattle livestock industries (defined in equation 1) by agricultural state district, with 0 being the lowest risk and 1 being the highest. [file 8892037.f1.docx]
